# Identification of a Quorum Sensing System Regulating Capsule Polysaccharide Production and Biofilm Formation in *Streptococcus zooepidemicus*

**DOI:** 10.3389/fcimb.2019.00121

**Published:** 2019-04-18

**Authors:** Zhoujie Xie, Kai Meng, Xiaoli Yang, Jie Liu, Jie Yu, Chunyang Zheng, Wei Cao, Hao Liu

**Affiliations:** ^1^MOE Key Laboratory of Industrial Fermentation Microbiology, College of Biotechnology, Tianjin University of Science and Technology, Tianjin, China; ^2^Tianjin Engineering Research Center of Microbial Metabolism and Fermentation Process Control, Tianjin University of Science and Technology, Tianjin, China; ^3^Robustnique Corporation Ltd., Tianjin, China

**Keywords:** quorum sensing, capsule polysaccharide, hyaluronic acid, biofilm, *Streptococcus zooepidemicus*

## Abstract

*Streptococcus zooepidemicus* is an important opportunistic pathogen of several species including humans. This organism is also well-known as the main producing strain in industrial production of hyaluronic acid (HA), which is the component of its capsule polysaccharide. How its virulence and capsule polysaccharide production is regulated remains poorly understood. Intercellular chemical signaling among bacteria provides communities of microbes the opportunity to coordinate gene expression to facilitate group behavior, such as pathogenicity, capsule polysaccharide production, etc. Yet no conserved cell-to-cell signaling system has been elucidated in *S. zooepidemicus*. Encoded within the genome of *S. zooepidemicus* is one Rgg regulator encoding gene (*rgg*) with low similarity to both *rgg2* and *rgg3* from *Streptococcus pyogenes*. A small ORF (named as *shp*) encoding a novel short hydrophobic peptide (SHP) was found in the vicinity of *rgg*. We found that the active form of pheromone is short and hydrophobic (LLLLKLA), corresponding to the C terminal 7 amino acids of the pre-peptide Shp, which shows divergent sequence to all peptide pheromones reported in streptococci. In response to active SHP, Rgg functions as a transcriptional activator to induce the expression of *shp*, forming a positive feedback circuit. Bacteria social behaviors, such as capsule polysaccharide production and biofilm formation, were significantly affected when the *rgg*-*shp* pathway was inactivated. These data provide the first demonstration that Rgg/Shp signaling pathway comprises an active quorum sensing system in *S. zooepidemicus*.

## Introduction

*Streptococcus equi* subsp. *zooepidemicus* (commonly referred to as *Streptococcus zooepidemicus*) is an important opportunistic pathogen of several species, notably equines and sometimes humans (Waller, 2017). This microorganism generally inhabits the mucous membranes and skin of healthy animals, and causes severe diseases such as pneumonia, septicemia, or meningitis in some conditions. It is a rare cause of human invasive disease originating from zoonotic transmission from domesticated animals (Pelkonen et al., 2013). Why this bacterium causes persistent infection and disease under certain circumstances remains largely unknown. It is believed that the regulated expression of capsular polysaccharide in *S. zooepidemicus* is one of the essential factors for the pathogenicity and dissemination of this microorganism (Xu et al., 2016). The main component of capsular polysaccharide for *S. zooepidemicus* is hyaluronic acid (HA), which is a component that is widely used in ophthalmic surgery and as an ingredient in cosmetics. This character also makes *S. zooepidemicus* the first HA commercial producer on an industrial scale, and remain the major strain in the industrial production of HA till now (Liu et al., 2011). Capsular polysaccharide production is usually a social behavior of bacteria regulated by cell-cell communication. For example, capsular polysaccharide synthesis and virulence in *Staphylococcus aureus* are controlled by an AI-2 quorum-sensing (QS) system (Zhao et al., 2010), and extracellular polysaccharide production in *Streptococcus pneumoniae* is regulated by a small hydrophobic peptide (SHP) QS system (Junges et al., 2017). Despite the clinical significance and economic importance of *S. zooepidemicus*, the identity of a widespread, conserved intercellular signaling system remained elusive in this organism.

QS is a widespread cell-cell signaling system among bacteria, that generate population-wide responses to low molecular weight signaling molecules (also referred to as pheromones), depending on cellular density (Miller and Bassler, 2001). QS provides communities of microbes the opportunity to coordinate gene expression to facilitate group behavior, such as virulence, extracellular polysaccharide production and biofilm formation (Rutherford and Bassler, 2012). For most QS signaling in Gram-negative bacteria, a small molecule such as N-acyl homoserine lactone is used as the secreted pheromone (Papenfort and Bassler, 2016), while for QS in many species of Gram-positive bacteria, secreted short peptide signals were usually sensed by protein receptors that alter the expression of downstream target genes (Lyon and Novick, 2004). Rgg QS pathways, widely found among low G+C Gram-positive bacteria, use Rgg transcription factors as cytoplasmic pheromone receptors for SHP pheromones (Fleuchot et al., 2013). Genome-wide surveys showed that such systems are present in the majority of streptococci (Fleuchot et al., 2011). Among streptococci, *Streptococcus pyogenes* (Group A *Streptococcus*, GAS) is perhaps the best understood species in terms of the role the Rgg QS (Jimenez and Federle, 2014). The gene for the SHP pheromone (*shp*) and the gene for Rgg transcription factor (*rgg*) have a characteristic arrangement in the genome with *shp* being located in the vicinity of *rgg* in GAS. Interestingly, different Rgg regulators might exhibit diverse regulatory effects. For example, there are two Rgg protein family members (Rgg2 and Rgg3) as cytoplasmic receptors of SHP pheromones in all completely sequenced GAS genomes. In response to the SHP pheromones, Rgg2 functions as a transcription activator, while Rgg3 functions as a transcription repressor (Chang et al., 2011).

*S. zooepidemicus* and GAS are phylogenetically related. Both of them belong to the pyogenic group of streptococci (Kawamura et al., 1995). However, there is no report that any Rgg protein functions as a QS effector in *S. zooepidemicus*. Here we demonstrate for the first time that an Rgg family transcriptional regulator in *S. zooepidemicus* functions as QS effector protein in response to a novel peptide pheromone (SHP) encoded by a neighboring *shp* gene. We provide further evidence that the Rgg transcriptional regulator functions as transcription activator to induce the expression of *shp*, and regulates HA production and biofilm formation. Therefore, Rgg/Shp comprises the first characterized quorum-sensing pathway in *S. zooepidemicus*.

## Materials and Methods

### Bacterial Strains, Plasmids, and Culture Conditions

All plasmids and strains used in this study are listed in Table 1. All *Streptococcus* strains were grown in Todd-Hewitt medium (Difco) supplemented with 0.3% (wt/vol) yeast extract or in Chemically Defined Media (CDM) (van de Rijn and Kessler, 1980). All *Streptococcus mutans* strains were grown anaerobically (in a Candle Extinction Jar) at 37°C. All *Streptococcus zooepidemicus* strains were grown under aerobic conditions at 30°C or 37°C. When testing for HA production, cells were grown in a THYE containing 0.2% sucrose. For the selection of antibiotic-resistant colonies in *S. mutans*, THYE plates were supplemented with spectinomycin (Sigma) (1,000 μg ml^−1^). For the selection of antibiotic-resistant colonies in *S. zooepidemicus* THYE plates were supplemented with spectinomycin (Sigma) (200 μg ml^−1^).

**Table 1:** Bacterial strains and plasmids used in this study.

**Plasmid or strain**	**Genotype/phenotype^a^**	**References; character**
**PASMIDS**		
pDL278	*E.coli*-*Streptococcus* shuttle vector,Spec^r^	LeBlanc et al., 1992
pSET4s::*sacB*	*Streptococcus* temperature sensitive plasmid with *sacB* as a counter-selection marker, Spec^r^	Sun et al., 2013
pZX9	A plasmid containing luciferase gene, Spec^r^	Xie et al., 2013b
pRPluc	pDL278::*rgg*::P*shp*::*luc*, Spec^r^	Reporter plasmid
pPluc	pDL278::P*shp*-*luc*, Spec^r^	Reporter plasmid
pSLR	pSET4s::*sacB*::*shp*LR, Spec^r^	Used for Δshp construction
pRSLR	pSET4s::*sacB*::*rgg-shp*LR, Spec^r^	Used for Δrgg-shp construction
**STRAINS**		
SZE	*S. equi* subsp. *zooepidimicus* ATCC35246, wild type strain	Ma et al., 2011
UA159	*S. mutans* UA159,wild type strain	Ajdić et al., 2002
159RPluc	UA159/pRPluc, Spec^r^	Test bed assay
	UA159/PDL278::P*shp*-*luc*	Test bed assay
Δshp	SZE, Δshp, markerless	*shp* deletion mutant
Δrgg-shp	SZE, Δ*rgg*,Δ*shp*, markerless	*rgg*-*shp* double deletion mutant
WTPluc	SZE/pPluc, Spec^r^	SZE harboring pPluc
RPluc	Δrgg-shp/pRPluc, Spec^r^	Δrgg-shp harboring pRPluc
Pluc	Δrgg-shp/pPluc, Spec^r^	Δrgg-shp harboring pPluc

a *Spec^r^, spectinomycin resistance*.

### Construction of Firefly Luciferase Reporter Plasmids

Primers used in this study are listed in Table 2. For Plasmid pRPluc (pDL278 vector containing a luciferase open reading frame driven by *shp* promoter and an intact *rgg* gene)construction, the fragment containing the *rgg* gene and the full intergenic region between *rgg* and *shp* was generated by PCR with the primer pair 763F/shpR from the genome of *S. zooepidemicus*, and luciferase open reading frame (*luc*) was amplified with primer pair lucF/R from plasmid pZX9 (Xie et al., 2013b). There are overlapping regions between the two amplicons, which allowed a subsequent overlapping PCR using primer pair 763F and lucR. The resulting 3-kb amplicons were digested with *Bam*HI and *Eco*RI and ligated into the corresponding sites of pDL278 to obtain pRPluc. For Plasmid pPluc (pDL278 vector containing a luciferase open reading frame driven by *shp* promoter)construction, *shp* promoter was generated by PCR with the primer pair shpF/shpR, and luciferase open reading was amplified with primer pair lucF/R. There are overlapping regions between the two amplicons, which allowed a subsequent overlapping PCR using primer pair shpF and lucR. The resulting 1.6-kb amplicons were digested with *Bam*HI and *Eco*RI and ligated into the corresponding sites of pDL278 to obtain pPluc.

**Table 2:** Primers used in the study.

**primer**	**Sequence 5^**′**^ → 3^**′**^**	**Purpose**
763F	GGAATTCCCACGAACGAAAATCA AGCTT	pRPluc construction
shpR	TGCTAAAAATATGTTGAACAATGG AAGACGCCAAAAACAT	pRPluc construction pPluc construction
lucF	ATGTTTTTGGCGTCTTCCATTGTT CAACATATTTTTAGCA	pRPluc construction pPluc construction
lucR	CGGGATCCCGAAACCTCCAAAA AATTATGT	pRPluc construction, pPluc construction
shpF	GGAATTCCCACGAACGAAAATC	pPluc construction
rggdnF	AGCTCTTTTCTCTTTTCAAGCCC TAAAAAACTGATTT	Δrgg-shp construction
rggdnR	CGGGATCCCGCTGCTTAGTGAT GTGCCT	Δrgg-shp construction
shpdnF1	AAATCAGTTTTTTAGGGCTTGAAA AGAGAAAAGAGCT	Δrgg-shp construction
shpdnR	GGAATTCCTGGTCTGGTTATTGAT GACGAGC	Δrgg-shp construction, Δshp construction
shpupF	CGGGATCCCGGCATTTACAG ATAAAGCC	Δshp construction
shpupR	AGCTCTTTTCTCTTTTCATGTTC AACATATTTTTAGC	Δshp construction
shpdnF2	GCTAAAAATATGTTGAACATGAAA AGAGAAAAGAGCT	Δshp construction

### Transformation of *S. mutans* and *S. zooepidemicus*

*S. mutans* was transformed using the natural transformation assay as previously described (Xie et al., 2013a). *S. zooepidemicus* was transformed using electroporation method as previously described (Sun et al., 2013).

### Construction of *shp* Deletion Mutant and *rgg*-*shp* Double Deletion Mutant

The *shp* gene deletion mutant (Δ*shp*) was constructed using a markerless gene-deletion system as described previously (Sun et al., 2013). Briefly, using *S. zooepidemicus* genomic DNA as the template, the upstream and downstream fragments of *shp* were amplified by PCR with primer pair shpupF/R and shpdnF2/R, respectively. There are overlapping regions between the two amplicons, which allowed a subsequent overlapping PCR using primer pair shpupF and shpdnR. The resulting amplicons were digested with *Eco*RI and *Bam*HI and ligated into the corresponding sites of SET4s::*sacB* to obtain pSLR. pSLR was transformed into *S. zooepidemicus* wild type, and the resulting transformant was first grown at 30°C for 12 h and then further cultured at 37°C for another 4 h in THYE medium supplemented with 200 μg/mL spectinomycin to select for the single crossover strain. After that, the single crossover strain was cultivated in THYE medium without antibiotics addition. Thereafter, the sucrose-resistant strains were selected on THYE plate supplemented with 5% (w/v) sucrose. Finally, the sucrose-resistant and spectinomycin-sensitive clones were isolated, and *shp* deletion mutants were examined by PCR and further confirmed by sequencing. The same strategy was used for the construction of *rgg*-*shp* double deletion mutant (Δrgg-shp). During the construction of Δrgg-shp, the primer pair rggdnF/R and primer pair shpdnF1/R were used, and plasmid pRSLR was constructed. The primers used for the construction of gene deletion mutants are listed in Table 2.

### Preparation of Synthetic Peptides

The peptides used in this experiment were purchased from GenScript (Nanjing, China) and the purity of all peptides were above 98%. Stock solutions were dissolved in DMSO or some weak acid according to the instructions at a concentration of 1 mM. Peptide sequences are the following, sSHP-C6: LLLKLA; sSHP-C7-1: LLLLKL; sSHP-C7: LLLLKLA; sSHP-C8: DLLLLKLA; sSHP-C9: HDLLLLKLA; sSHP-CN: LPYFAGCL.

### *S.mutans* “Test-Bed” Reporter Assay

Reporter plasmids pRPluc and pPluc were transformed into *S.mutans* UA159 respectively, to obtain *S.mutans* 159RPluc and 159Pluc, respectively. The luciferase activity in response to synthetic peptides in each reporter strain was detected based on a previously described method (Shanker et al., 2016). Briefly, the reporter strain was incubated overnight in 5 ml of CDM, which were supplemented with spectinomycin (100 μg/μl). The overnight culture was diluted in multiples into fresh CDM until the OD600 reached to 0.1, then added different concentrations of the test peptide to make the final concentration of the peptide in the culture was 0, 5, 10, 25, 50, 100, and 150 nM. Thereafter, the cultures were continued to be incubated under anaerobic conditions at 37 ° C for 1 h. Finally, a 180 μl aliquot of each culture was placed in a Falcon white flat-bottom 96-well plate with the addition of 20 μl of 1 nM D-luciferin sodium salt. The optical density (OD) at 600 nm and luminescence of the culture were measured using an Infinite 200PRO multi-plate reader. Normalized luciferase activity was expressed as relative light units (RLU) (luminescence value)/*OD*_600_.

### Growth Curve and Time Course Luciferase Activity of Wild-Type *S. zooepidemicus* Containing Plasmid pPluc

Reporter plasmids pPluc was transformed into wild-type *S. zooepidemicus* to obtain strain *S. zooepidemicus* WTPluc. The reporter strain WTPluc was grown at 37°C in CDM after dilution from the overnight culture to an *OD*_600_ of 0.01. The growth and luciferase activity of the reporter strain were monitored throughout growth by taking 180 μl aliquots every 4 h from the growing cultures. Aliquots were placed into a Falcon white flat-bottom 96-well plate and were mixed with 20 μl of 1nM D-luciferin. After that, the *OD*_600_ and Luminescence were measured using an Infinite 200PRO multi-plate reader. Normalized luciferase activity was expressed as relative light units (RLU) /*OD*_600_.

### Luciferase Transcriptional Reporter Assay in *S. zooepidemicus*

Reporter plasmids pRPluc and pPluc were transformed into *S. zooepidemicus* Δrgg-shp to obtain *S. zooepidemicus* RPluc and Pluc, respectively. The luciferase activity in response to synthetic peptide (sSHP-C7, at a final concentration of 100 nM) for each reporter strain was measured as the method described above in *S.mutans* “test-bed” reporter assay.

### HA Production Determination

Exopolysaccharide produced by *S. zooepidemicus* was identified and confirmed as HA previously (Vázquez et al., 2015). For exploring the effect of *rgg*-*shp* pathway on the exopolysaccharide production in *S. zooepidemicus, S. zooepidemicus* wild type and pathway deletion mutant Δrgg-shp were cultivated in THYE medium containing 0.2 % sucrose with agitation (200 rpm) at 37°C. Samples were taken at 2, 4, 6, 8, 12, 16, and 24 h, respectively. HA concentration for each sample was determined by CTAB Turbidimetric method as described previously (Chen and Wang, 2009). Briefly, the sample culture was capsule-released by adding 0.1% (w/v) SDS and centrifuged at 10,000 g for 10 min to remove cells. The supernatant was mixed with three volumes of absolute ethanol and held at 4°C for 1 h. The precipitant was collected by centrifugation at 5,000 g for 10 min and re-dissolved in one volume distilled water. Two volumes of cetyltrimethyl-ammoniun bromide (CTAB) buffer (2.5 g L^−1^) were added to the samples and mixed gently for a reaction time of 10 min at room temperature, followed by *OD*_600_ measurement. HA concentration was determined based on the *OD*_600_ and a standard curve, which was established using an HA stock solution prepared using standard HA (Sigma).

### Biofilm Assay

Biofilm assays were performed as described before (O'Toole, 2011; Yi et al., 2014), with some modifications. Briefly, bacterial strains were grown overnight in THYE medium at 37°C, then diluted 1:10 into fresh CDM containing 1% fibrinogen, and 0.6 ml volumes were aliquoted to three separate wells of a 96-well polystyrene plate. When indicated, synthetic peptide (sSHP-C7) was added to a final concentration of 100 nM. Plates were incubated at 37°C for 24 h without shaking. Medium alone served as a negative control. Biofilm production was determined using crystal violet staining. Briefly, the biofilm sample was visualized by staining with 1 % crystal violet for 10 min after washing with PBS. After adding 200 μl of 95% ethanol, *A*_595_ was measured by an Infinite 200PRO multi-plate reader. The experiment was repeated three times.

### Data Analysis

All data points shown in this study represent the average values from three independent experiments. Error Bars show standard deviations. Statistical analysis was performed by using the two-tailed Student's *t*-test. *P* < 0.05 was considered to be statistically significant. Statistical significance was concluded at ^*^P < 0.05, ^**^P < 0.01, ^***^P < 0.001.

## Results

### Presence of *rgg*-Like Gene in *S. zooepidemicus*

**Figure 1:**
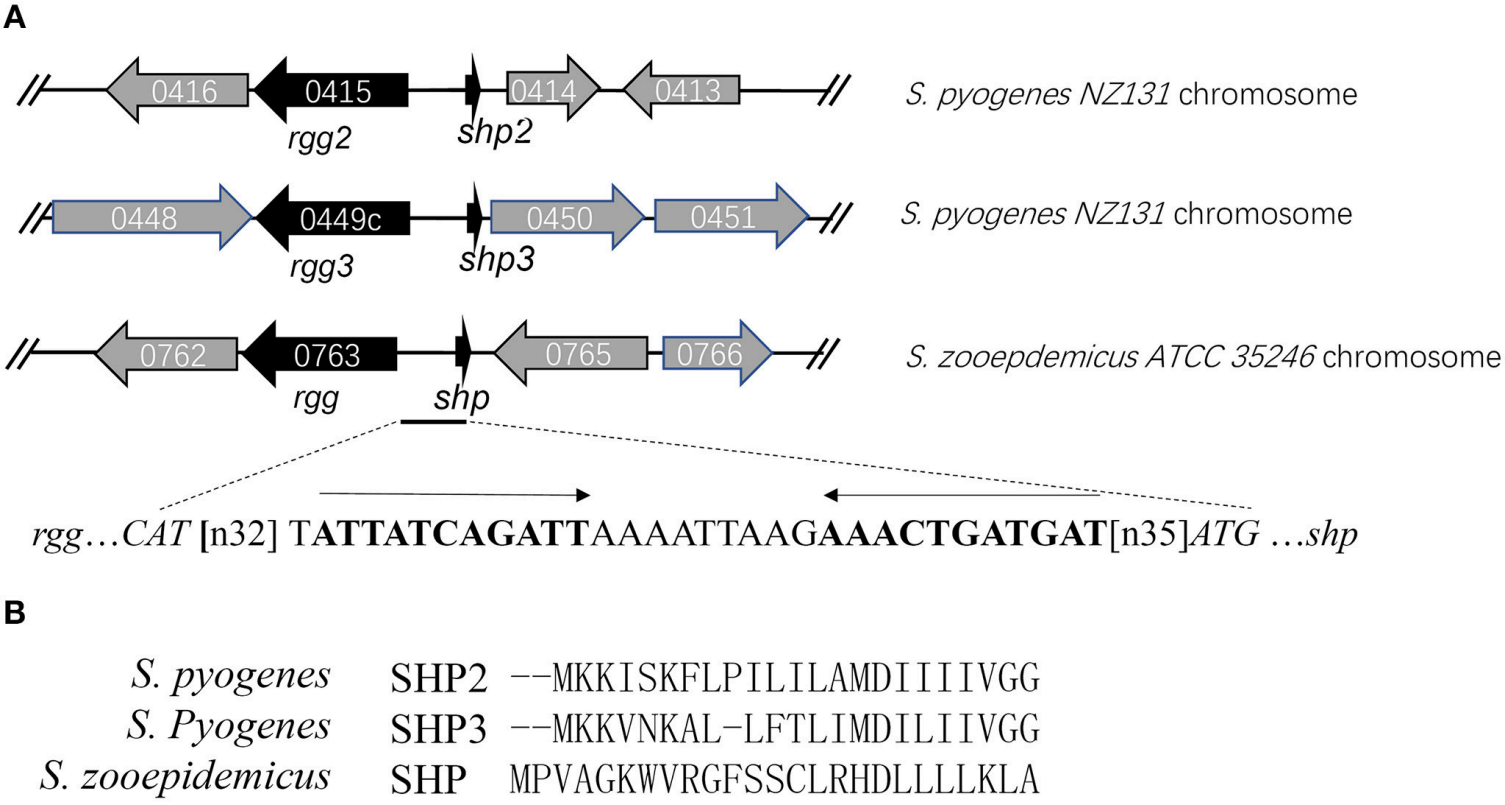
Rgg/SHP signaling system in the *S*. *zooepidemicus* genome. **(A)** The genetic organization of *rgg* and *shp*. The inverted repeat located in the intergenic region is shown in bold. **(B)** Alignment of pre-peptides SHP2, SHP3 (from *S. pyogenes*) and SHP (from *S. zooepidemicus*) indicating their sequence divergence.

The sequences of the characterized Rgg proteins (Rgg2 and Rgg3) from *S. pyogenes* were used as templates for homology searching in the genome of *S. zooepidemicus* ATCC 35246 (GenBank accession number: NC_017582). The best matches for both searches were found in the same gene (SeseC_00763). SeseC_00763 (designated as *rgg*) shows 31 and 30% identities to rgg2 and rgg3, respectively. Since most Rgg regulator genes are located close to their associated pheromone-encoding genes, a closer examination of the *rgg* locus was performed. We identified a 78-bp Open reading frame (ORF) in the vicinity of *rgg*, that was not annotated in the public database. The genomic coordinates of the small ORF are at bases 632,144 to 632,221. An invert repeat motif was found in the intergenic region between *rgg* and the small ORF, which is predicted to be the binding site of Rgg (Figure 1A). The small ORF encodes a 25-amino-acid hydrophobic peptide and was therefore referred to *shp*. Shp shows no sequence similarity with SHP2 and SHP3 from GAS (Figure 1B), whereas the putative product of *shp* meets the criteria of short hydrophobic peptides (SHPs) established by Ibrahim et al (Ibrahim et al., 2007). Furthermore, we performed homology searches in other strains of *S. equi* by using Rgg as a template and found that all the 15 fully sequenced strains of *S. equi* available in the NCBI database, including 10 stains of *S. equi* ssp *zooepidemicus* and 5 strains of *S. equi* ssp *equi*, carry an Rgg-homologous protein with more than 98% identity. This indicates that the system is widely conserved in different strains of *S. equi* species.

### The SHP Pheromone Acts as an Autoinducer via Rgg

The study results of Rgg quorum sensing pathways in other Gram-positive bacteria indicate that the SHP pheromones usually undergo post-translational processing steps to release the C-terminal five to eight amino acids of the peptide as the active pheromone, and that the active SHP pheromone frequently forms a positive feedback loop with the corresponding Rgg regulator, stimulating a fast response to activate (or repress) the expression of SHP encoding gene (Fleuchot et al., 2013). *S. mutans* has been successfully used as the heterologous expression host to study the interaction between different Rgg regulators and SHP pheromones from different species (Shanker et al., 2016). A similar assay was performed in our study to make a fast assessment on the response of Rgg to SHP peptide from *S. zooepidemicus*. The fact that *S. mutans* does not contain the *shp* homolog of *S. zooepidemicus* make it feasible to use *S. mutans* as the test bed.

**Figure 2:**
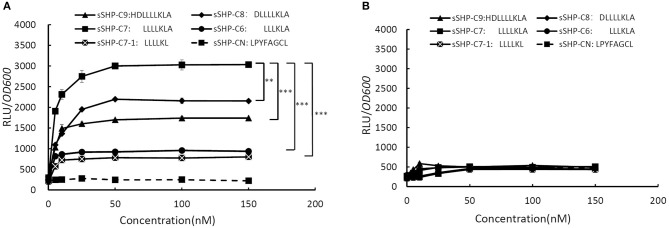
Effect of the sSHP pheromones on relative expression of *shp* promoter (P*shp*) in the *S. mutans* test-bed assay. **(A)** Relative Luciferase activities (RLU/*OD*_600_) of strain 159RPluc (*Streptococcus mutans* UA159 containing plasmid pRPluc) in response to increasing concentrations of synthetic peptides derived from the C-terminal region of Shp (sSHP-C6, sSHP-C7-1, sSHP-C7-1, sSHP-C8, sSHP-C9) and negtive-control peptide (sSHP-CN). **(B)** Relative Luciferase activities (RLU/*OD*_600_) of strain 159Pluc (*Streptococcus mutans* UA159 containing plasmid pPluc) in response to increasing concentrations of synthetic peptides derived from the C-terminal region of Shp and negtive-control peptide (sSHP-CN). All data points shown in this figure represent the average values from three independent experiments. Error Bars show standard deviations. **P < 0.01, ***P < 0.001 (2-tailed paired *t*-test).

To do the *S. mutans* test bed experiment, we firstly constructed two luciferase transcriptional reporter plasmids pPluc and pRPluc. In the plasmid pPluc, the full intergenic region between *rgg* and *shp* from *S. zooepidemicus* is fused with fire fly luciferase gene *luc*, which is driven by the promoter of *shp* (P*shp*). The structure of pRPluc is similar as pPluc, except that an intact *rgg* is included in pRPluc. These two reporter plasmids were transferred into *S.mutans* UA159 to obtain strain 159Pluc and 159RPluc respectively, allowing us to test whether the Rgg could regulate the expression of P*shp* in response to added synthetic SHP peptides. Based on the sizes of known SHP pheromones from other bacteria, we synthesized five peptides corresponding to truncated C-terminal derivatives of Shp. An SHP from *S. thermophilus* (LPYFAGCL) was also synthesized as a negative-control peptide (Fontaine et al., 2010). The peptides were tested in chemically defined medium (CDM), which contains individual amino acids instead of polypeptides. Synthetic peptides were added to low-density log-phase cultures of *S.mutans* 159Pluc and 159RPluc. Luciferase activity was then measured 60 min thereafter. As shown in Figure 2A, all synthetic peptides, except the control peptide (sSHP-CN) from *S. thermophilus*, could activate P*shp* in a dose dependent manner in the reporter strain of 159RPluc, while the synthetic peptide corresponding to the C-terminal 7 amino acids of Shp (sSHP-C7) exhibited the strongest induction in reporter activity. P*shp* was induced by sSHP-C7 at the concentration as low as 5 nM, and approached a maximum rate at 100 nM, suggesting that sSHP-C7 is, or closely resembles, the mature form of SHP pheromone. As shown in Figure 2B, no induction in reporter activity was observed in the reporter strain of 159Pluc, indicating the reporter induction is dependent on the presence of Rgg. Taken together, the results of the test-bed assay indicate that *shp* from *S. zooepidemicus* encodes an active pheromone which acts as an autoinducer via Rgg, and that Rgg functions a transcription activator in response to the active SHP pheromone to activate the expression of *shp*.

### The SHP Pheromone Acts as an Autoinducer in *S. zooepidemicus*

**Figure 3:**
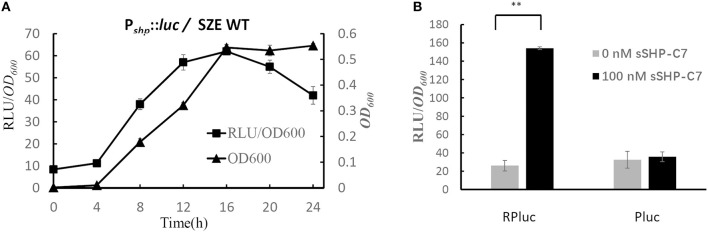
Rgg and Shp comprise an active autoinducing system in *S. zooepidemicus*. **(A)** The graph shows the bacterial growth (*OD*_600_) and relative luciferase activities (RLU/*OD*_600_) for strain WTPluc (wild-type *S. zooepidemicus* containing plasmid pPluc). The data shown are average values from three independent experiments ± standard deviations. **(B)** Relative Luciferase activities (RLU/*OD*_600_) of strain RPluc (*S. zooepidemicus*Δrgg-*shp* containing pRPluc) and Pluc (*S. zooepidemicus*Δrgg-shp containing pPluc) in response to synthetic peptide (0 nM and 100 nM). The data shown are average values from three independent experiments. Error Bars show standard deviations. **P < 0.01 (2-tailed paired *t*-test).

The results of test bed experiment indicate that Rgg/Shp comprises an active qurom sensing system. In order to confirm it is the real case in *S. zooepidemicus*, we transformed the plasmid pPluc into *S. zooepidemicus* wild type strain. The resulting strain WTPluc were grown in CDM at 37°C, and cell density and luciferase activity were monitored throughout growth. The results presented in Figure 3A show that the expression of P*shp* was low in the lag phase, and induced constantly with the increase of the OD value in the log phase. The maximum RLU/*OD*_600_ was obtained in the late-log phase and remained high when the culture entered into the stationary phase. The typical QS-regulated expression pattern of P*shp* suggests that *shp* is regulated by a QS system in *S. zooepidemicus*.

To provide direct evidence that Rgg/Shp comprise an active quorum sensing system in *S. zooepidemicu, rgg*/*shp* double deletion strain (Δrgg-shp) was constructed using the marker-less genetic manipulation system we established before (Sun et al., 2013). Luciferase reporter plasmids (pRPluc and pPluc) were transformed into Strain Δrgg-shp, respectively. The resulting strains RPluc and Pluc were cultured in CDM. Luciferase activities were measured for cultures treated with the predicted mature pheromone (sSHP-C7) and for untreated cultures. As shown in Figure 3B, gene expression of P*shp* was significantly induced in cultures treated with the specific pheromone for strain Δrgg-shp containing pRPluc. While the addition of the synthetic pheromone into the strain Pluc (Δrgg-shp containing pPluc) showed no upregulation of P*shp* expression compared with the untreated samples. These results indicated that Rgg/Shp is indeed a functional autoinducing system in *S. zooepidemicus*. Altogether, our results demonstrate that Shp is the precursor of the active pheromone, and that Rgg acts as an activator by responding to the pheromone to activate the expression of *shp*, forming a positive feedback regulation loop in *S. zooepidemicus*.

### Inactivation of the Rgg/Shp System Decreases Capsule Polysaccharide Production and Biofilm Formation

**Figure 4:**
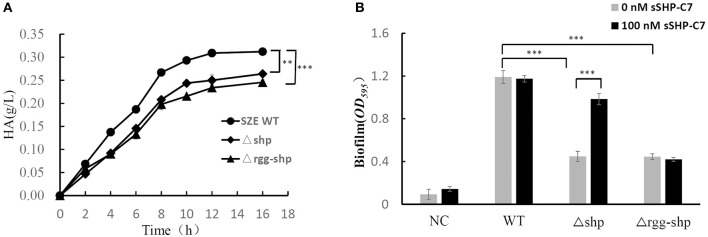
Rgg/Shp signaling pathway plays a key regulatory role in important social behavior processes of *S. zooepidemicus*. **(A)** HA production time course data from the wild-type *S. zooepidemicus* (SZE WT),deletion mutants Δrgg-shp and Δshp in flask cultures. **(B)** Biofilm formation of wild-type *S. zooepidemicus* (SZE WT), deletion mutants Δrgg-shp and Δshp when treated with 0 or 100 nM synthetic peptide, respectively. The medium without inoculation was used as the negative control (NC). All data points shown in this figure represent the average values from three independent experiments± standard deviations. **P < 0.01, ***P < 0.001 (2-tailed paired *t*-test).

With the evidence that the *rgg*/*shp* system provides *S. zooepidemicus* with the capability to communicate intercellularly, we next asked what social behavior does the Rgg/Shp system regulate in *S. zooepidemicus*. For this purpose, capsule polysaccharide (CPS) production and biofilm formation were tested and compared for wild-type and *rgg*/*shp* deletion mutants. To test if the Rgg/SHP signaling pathway is involved in the production of CPS, which is composed with HA in the case of *S. zooepidemicus*, HA production throughout the fermentation process was detected for wild type stain (SZE WT), *shp* single deletion strain (Δshp) and *rgg*-*sh*p double deletion strain (Δrgg-shp). As shown in Figure 4A, both *shp* single deletion and *rgg*-*shp* double deletion result in lower production of HA. The final titer of Δshp and Δrgg-shp is similar, and is about 20% lower than that of wild-type, indicating Rgg/Shp signaling pathway has a positive regulation on the production of capsule polysaccharide.

To detect whether the Rgg/SHP signaling pathway affects the ability of biofilm formation, biofilm formation abilities of the wild-type strain, *shp* single deletion strain(Δshp) and *rgg*/*sh*p double deletion strain(Δrgg-shp) were evaluated using a standard crystal violet staining method. As showed in Figure 4B, compared with the wild-type strain, deletion of *shp* results in very low production of biofilm, and *rgg/shp* double deletion strain exhibited similar result. We next asked if treatment with sSHP could restore the biofilm formation for those mutant strains. Biofilm formation abilities were assessed for the samples treated with 100 nM sSHP-C7. As shown in Figure 4B, exogenous addition of sSHP-C7 to wild-type did not cause an obvious change in biofilm formation; and *shp* deletion mutant partially restored the biofilm formation ability when sSHP-C7 was added, whereas no recovery was observed when the sSHP-C7peptide was added to *rgg*/*shp* double deletion strain. All these results indicate that Rgg/Shp signaling pathway played a positive regulatory role in the process of biofilm formation.

Taken together, our results demonstrate that the Rgg-shp system played a key regulatory role involved in important group processes, such as capsule polysaccharide production and biofilm formation.

## Discussion

Intercellular signaling communication among bacteria provides communities of microbes the opportunity to coordinate gene expression to facilitate group behaviors. An established paradigm for intercellular communication among streptococci relies on peptide signaling molecules and Rgg transcriptional regulator. The signaling peptide is ribosomally-synthesized as an unmodified, linear pre-peptide that is secreted and processed to an active pheromone, then imported to the cytoplasm and recognized by Rgg regulator that alters gene expression (Fleuchot et al., 2011; Neiditch et al., 2017). However, examples of widely conserved quorum-sensing systems have largely remained unknown for *S. zooepidemicus*. Here we present evidence for the presence of an active QS system which is composed of Rgg regulator and a peptide signaling pheromone (encoded by *shp*) in *S. zooepidemicus*. The predicted products of *shp* are composited of 25 amino acids. Our data support that the mature form of signaling peptide is corresponding to the last seven amino acids of the pre-peptide Shp. So, to fulfill its putative signaling function, pre-peptide Shp must be secreted and processed through some specific pathway that releases the mature pheromone. In signal-receiving cells, mature pheromone must be imported to the cytoplasm, where it directly engages and controls the activity of Rgg transcriptional regulator. The mechanism about how Shp is processed, exported and imported remains to be studied. It should be noted that the Rgg transcriptional regulator identified in this study is conserved in all sequenced genomes of *S. equi*, indicating that it is a conserved signaling system in the species of *S. equi*. To the best of our knowledge, this is the first demonstration of Rgg-SHP mediated cell-cell signaling in the species of *S. equi*. It is intriguing to point out that active signal peptide (LLLLKLA) identified in *S. zooepidemicus* shows divergent sequence with any known SHP pheromones in other species. It is tempting to speculate that the Rgg/Shp pathway identified in this study is involved in intra-species communication, not inter-species communication.

An emerging theme in social behaviors controlled by quorum sensing is the regulation of surface polysaccharide expression. The connection between quorum sensing and extracellular polysaccharide production has been reported in a wild range of bacteria, such as *S. pneumoniae* (Junges et al., 2017), *Staphylococcus aureus* and *Vibrio vulnificus* (Zhao et al., 2010; Lee et al., 2013). In this study, inactivation of *rgg*/*shp* system led to a decreased exopolysaccharide (HA) production, indicating the *rgg*/*shp* system has a positive regulatory role on the capsule HA production in *S. zooepidemicus*. Extracellular polysaccharides have long been known to affect biofilm formation in other bacteria (Vu et al., 2009; Limoli et al., 2015). Our study did show that *rgg*/*shp* system positively affects the production of biofilm in *S. zooepidemicus*, however at the current stage, we cannot conclude that *rgg*/*shp* system affects the biofilm formation through the regulation of the extracellular polysaccharide. Altogether, our data support that *rgg*/*shp* system positively regulates both capsule polysaccharide production and biofilm formation in *S. zooepidemicus*. Although the direct target of the Rgg/Shp signaling pathaway and its mechanism in the regulation of exopolysaccharide production and biofilm formation remains unknown at current stage, we have provided the groundwork for studies aimed at revealing the role of *rgg*/*shp* system in coordinating bacterial social behavior in *S. zooepidemicus*.

An increased understanding of how cell-cell signaling systems work is leading to new possibilities for manipulating bacterial functions. In the clinical area, some interesting studies have been conducted recently to target QS as a tool to combat or modulate infections associated with bacterial biofilms (Aggarwal et al., 2015; Ishii et al., 2017). In biotechnology area, QS in bacteria has been applied for induction of recombinant protein expression (Tsao et al., 2010), dynamical regulation of the expression of target genes (Gupta et al., 2017), microbial biosensors designing, etc (Choudhary and Schmidt-Dannert, 2010). So far as we know, our study is the first demonstration of Rgg/SHP mediated cell-cell signaling in *S. zooepidemiccus*, the significant streptococci species both in the clinical area and in the industrial area. Our finding is of importance for the design of quorum sensing inhibitory strategies in controlling *S. zooepidemiccus* infection, and is also of importance for metabolic engineering of *S. zooepdemicus* in commercially producing HA products by providing an autoinducing regulation module.

## Data Availability

All datasets generated for this study are included in the manuscript and/or the supplementary files. The datasets generated for this study can be found in GenBank, accession number: NC_017582.

## Author Contributions

ZX, HL, and WC designed the experiments. ZX, KM, XY, JL, and JY conducted most of the studies. CZ took part in HA production determination and data analysis. ZX, HL, and WC analyzed data. ZX, HL, and WC wrote the paper with the contributions from all other authors.

### Conflict of Interest Statement

The authors declare that the research was conducted in the absence of any commercial or financial relationships that could be construed as a potential conflict of interest.
